# A worksite prevention program for construction workers: design of a randomized controlled trial

**DOI:** 10.1186/1471-2458-10-336

**Published:** 2010-06-14

**Authors:** Karen M Oude Hengel, Catelijne I Joling, Karin I Proper, Birgitte M Blatter, Paulien M Bongers

**Affiliations:** 1 Body@Work TNO VUmc, Research Center Physical Activity, Work and Health, Amsterdam, the Netherlands; 2 TNO Quality of Life | Work & Employment, Hoofddorp, the Netherlands; 3 Department of Public and Occupational Health, The EMGO Institute for Health and Care Research, VU University Medical Center, Amsterdam, The Netherlands

## Abstract

**Background:**

A worksite prevention program was developed to promote the work ability of construction workers and thereby prolong a healthy working life. The objective of this paper is to present the design of a randomized controlled trial evaluating the effectiveness of that intervention program compared with usual care for construction workers.

**Methods:**

The study is designed as a randomized controlled trial with a follow-up of one year. Employees eligible for this study are construction workers performing actual construction work. The worksite intervention will be compared with usual care. This intervention was developed by using the Intervention Mapping approach and consists of the following components: (1) two individual training sessions of a physical therapist to lower the physical workload, (2) a Rest-Break tool to improve the balance between work and recovery, and (3) two empowerment training sessions to increase the influence of the construction workers at the worksite. Outcome measures are assessed at baseline, 3, 6, and 12 months. The primary outcome measures of this study are work ability and health-related quality of life. Secondary outcome measures include need for recovery, musculoskeletal complaints, work engagement and self efficacy. Cost-effectiveness will be evaluated from the company perspective. Moreover, a process evaluation will be conducted.

**Discussion:**

The feasibility of the intervention and the study has been enhanced by creating an intervention program that explicitly appeals to construction workers and will not interfere too much with the ongoing construction. The feasibility and effectiveness of this worksite prevention program will be investigated by means of an effect- and a process evaluation. If proven effective, this worksite prevention program can be implemented on a larger scale within the construction industry.

**Trial Registration:**

NTR1278

## Background

In order to face the challenges of the aging working population and to extend the healthy working lives of the workers, the construction industry in the Netherlands has reason to pay attention to maintaining and promoting work ability [1,2]. Work ability is defined as how well workers can perform their jobs at present and in the near future, and is the result of the interaction between the individuals' capacity and the work demands [3,4]. Work ability is determined by personal factors like health, functional capacity and job satisfaction and occupational factors like physical work demands and the work organization [4-6].

Because of the high physical workload and the health risks involved [7-10], construction workers run relatively high risks to suffer an impaired work ability [2,4]. To change things for the better, health promoting activities to maintain and improve the work ability seem necessary. Until now, most health promotion programs in the construction industry have focused on either improving the health of construction workers by means of a lifestyle program [11,12] or on decreasing the work demands by means of ergonomic measures [13,14]. Only one intervention study in the construction industry was found that explicitly aimed to improve the work ability. That single-component intervention consisting of a counseling and education program for construction workers at risk for disability pension showed a slight but not significant improvement of the work ability [15].

Based on the fact that work ability is a multidimensional concept, it was hypothesized that a multidimensional intervention approach could potentially be more effective. To our knowledge, such interventions have not yet been undertaken in the construction industry. Therefore, in our study a multidimensional intervention was developed, taking into account the individual factors as well as the work environment, in order to promote the work ability (Oude Hengel et al., Using intervention mapping to develop a worksite prevention program for construction workers, submitted). The intervention was developed by means of the Intervention Mapping approach which is based on theoretical information from literature and practical information from stakeholders [16]. This resulted in an intervention tailored to the needs of the construction workers. This paper presents the design of the worksite prevention program illustrating the recruitment of the workers, the feasibility of the study, and the attractiveness of the program for the workers.

## Methods

### Study design

A Randomized Controlled Trial is performed in order to evaluate the effectiveness of the intervention. This trial is carried out to evaluate whether the worksite prevention program for construction workers improves construction workers' work ability and their health-related quality of life. Construction workers at the worksites allocated to the intervention group receive the worksite prevention program during six months; those allocated to the control group receive no intervention (i.e. usual care). Participants are followed for one year. Primary and secondary outcomes are measured at baseline, and 3, 6 and 12 months after baseline measurement. The study protocol was approved by The Medical Ethics Committee of the VU University Medical Center (Amsterdam, The Netherlands).

### Study population

The study population consists of construction workers performing actual construction work (i.e., blue collar workers). These workers are contracted by six companies which are specialized in house-, commercial- or industrial building. The other inclusion criteria were (1) available for the study for the following 12 months, (2) sufficient mastery of the Dutch language and (3) having signed a written informed consent. No exclusion took place based on age or gender.

### Recruitment of the study population

In order to successfully accomplish an intervention program at the worksite, strong support and participation of different company levels (managers, supervisors and workers) was considered essential. At the start of the project, we therefore recruited the top-management of the six companies who committed themselves to the project by signing a letter of intent. Additionally, they agreed that their workers (supervisors and construction workers) were allowed to participate in the program during working hours. The managers informed all supervisors about the aim of the intervention and the intervention components. Finally, the researchers informed all workers at the worksite about the intervention program by an oral presentation and by handing out a letter with the content of the program.

### Randomization

Cluster randomization took place at the level of department within each company. In order to avoid intervention group contamination, to accommodate a potential work-related intervention, to obtain maximal cooperation of employers and employees, and to enhance participants' compliance, cluster randomization was considered the best randomization strategy for this study. The randomization was performed by a research assistant who had no prior information about the departments. For practical reasons, randomization was performed before the baseline measurements. Because the intervention takes place at the worksite, the participants, their supervisors and the trainers cannot be blinded to the group assignment.

### Intervention

The intervention was developed using the Intervention Mapping approach [16]. Intervention Mapping ensures participation and consultation of all stakeholders (employers, supervisors, workers, health professionals, and providers). The development of the detailed program plan was based on three key points: (1) feasibility: a program which could be executed at the worksite, not interfering too much with the ongoing construction work (2) attractiveness for workers: the program should be geared to the workers' perception of their work environment (3) a standardized protocol for a sound scientific evaluation. Based on the first step of Intervention Mapping, two program objectives were defined: (1) the program should improve the balance between the physical workload and the need for recovery and (2) the program should increase the range of influence of construction workers at the worksite. Following the steps of Intervention Mapping, the program objectives were transformed into an intervention program of six months. An extensive description of the Intervention Mapping process and the content of the intervention has been described elsewhere (Oude Hengel et al., Using intervention mapping to develop a worksite prevention program for construction workers, submitted). The intervention consists of a physical component and a mental component. At the start of the program, the intervention is introduced to the workers and their supervisors by a 3-minute lasting video showing the content of the intervention and the accompanying components. This video uses the metaphor of a soccer game to introduce the underlying principles of the intervention program.

The physical component consists of (1) two individual training sessions by a physical therapist and (2) a "Rest-Break Tool". To reduce the physical workload, the worker receives two training sessions by an occupational physical therapist. During the first training, the therapist assesses work style, working methods, and the balance between physical load and rest breaks, and makes an assessment of the associated health risks. This is done by means of a quick scan questionnaire and a 15-20 minute observation of the worker. Based on the assessment, the therapist gives individual advice on how to reduce the physical workload, focusing on the improvement of the work style, the work methods and/or the rest breaks. At the end of the first training session, the physical therapist writes down a maximum of three recommendations for the worker on a pocket-size card. Before the training session begins, the therapist meets the supervisor of the worksite to inform him about the purpose of his visit. After training all participating workers at one site, the physical therapist meets the supervisor in order to discuss the group results. During the second visit, after four months, the therapist discusses the experiences so far and evaluates the impact of the advice of the first training with the worker. If necessary, the physical therapist and the worker adjust the advice. Second, a Rest-Break Tool was developed that focuses on fatigue and need for recovery. The aim of the Rest-Break Tool is to raise awareness about the importance of reducing fatigue among workers by taking flexible rest breaks and to stimulate to actually take rest breaks in order to reduce fatigue. The Rest-Break tool was set up as a flowchart and consists of four steps: (1) the expectations of the workers about their own fatigue at the end of the working day, (2) short term advice to take mini rest breaks (20-60 seconds) or an additional break of ten minutes, (3) selection of possible causes of fatigue and (4) long term advice about structurally lowering fatigue. The Rest-Break Tool is introduced and explained to the workers by the therapist during the first visit. The workers are asked to fill in the tool weekly, alone or with colleagues, and to discuss the results with their supervisor. At the start of the program, the supervisors receive a folder with the Rest-Break Tools to hand out to the workers at a fixed time each week. A text message by the mobile phone is sent weekly as a reminder to all supervisors.

For the mental component of the intervention, workers receive two interactive empowerment training sessions. Due to practical reasons, the duration of the training is limited to one hour. The empowerment trainer is present at the worksite before the training to get an impression of the worksite. The training is aimed at improving the range of influence of the workers at the worksite. The workers are taught how to change their attitude from a passive towards a more proactive attitude by increasing the self-efficacy regarding (1) taking responsibility for their own health, (2) discussing with colleagues about the responsibility for their own behavior (e.g., taking rest breaks, asking for assistance during lifting tasks) and (3) improving the communication with the supervisor. A proactive attitude supposes that workers control and change possible adverse working conditions by themselves. The training consists of five components; (1) an introduction of the concept of self-efficacy within the construction industry, (2) an introduction of the training as part of the program, (3) an explanation about how to change, in general, a passive attitude towards a more proactive and positive attitude, (4) a list of topics (e.g., good teamwork, more communication with supervisor, more rest breaks) workers would like to change during the intervention, and (5) an action plan written down on a poster. The training is tailor-made which means that the five steps are just a rough outline of the training. The therapist meets the supervisor before the training sessions, to inform him about the purpose of his visit and to invite him to (partly) attend the meeting. After four months, during a follow-up meeting, the empowerment trainer and workers discuss, evaluate and reconsider the action plan and results that are already booked.

**Figure 1:**
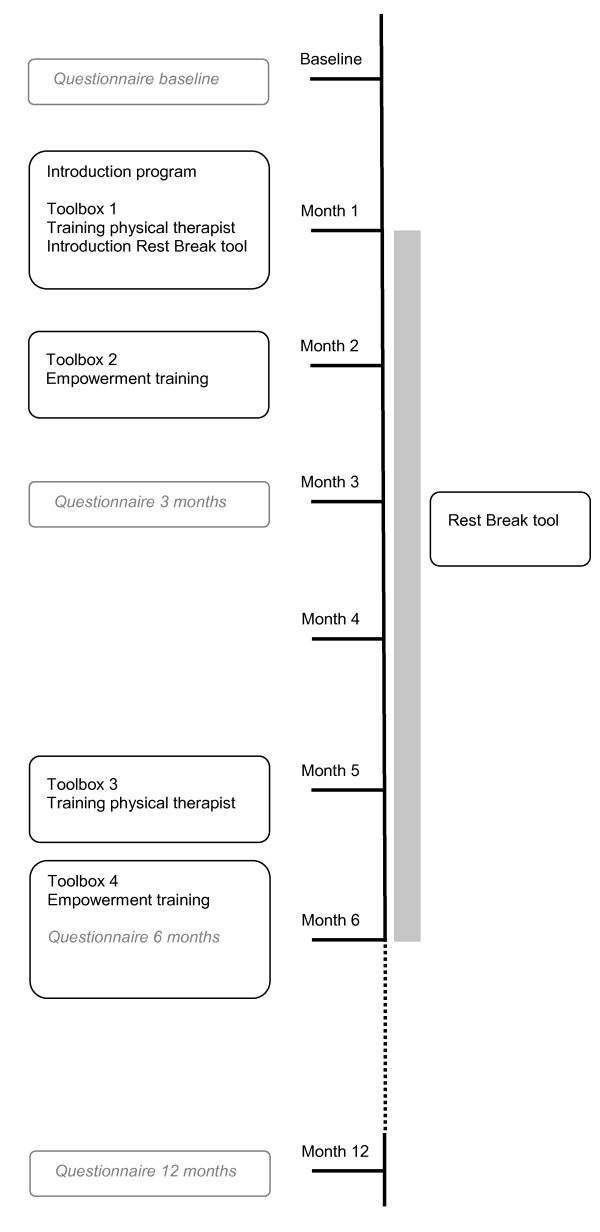
**Flow chart of the intervention program**.

Figure 1 presents the timeline of the current study including the different intervention components and the measurements. For feasibility reasons, all training sessions are organized within the existing so-called "toolbox education system" in the construction industry. The toolbox education system consists of at least 10 obligatory health and safety training sessions for workers, which have to be organized by employers in the construction industry each year. These training sessions are necessary in the construction industry to obtain an official safety and health certificate.

### Co-interventions

It is pointed out to the companies that participation in other intervention studies or programs aimed at health promotion (e.g. lifestyle programs, adjustments of the equipment, organizational changes) is not allowed during this study. At 12 months follow-up, managers are asked if any other intervention took place during the period of the current study. Some other health care use, like visiting a physical therapist, is regarded as usual care.

### Compliance and loss to follow up

All participants in this study receive a questionnaire at baseline and a follow-up at 3, 6 and 12 months after baseline measurement. Participants that withdraw from the intervention program are followed and receive the questionnaire at the given time points. To register the reasons for withdrawal, all participants are asked if they voluntarily want to give their reason(s) for discontinuing the intervention.

To minimize loss-to-follow up, the researchers distribute and collect the questionnaires at the worksite. In case of absence from work of the participants, the supervisors at the worksite are asked to hand out the questionnaire to the participants later on and to encourage the participants to complete the questionnaire and to send the questionnaire back in a stamped and addressed envelope. If the questionnaire is not received within three weeks, a new questionnaire is sent to the worker.

### Incentives

It is well-known that maintaining participants is a difficult process in intervention studies [17]. Therefore, incentives are distributed among the participants to make participation more attractive and to minimize loss to follow up. After the first empowerment training the participants receive a mug with the study logo. All participants receive playing cards with the study logo after the second questionnaire (at three months follow-up). Moreover, as a reminder of the project, posters are distributed after the first and second empowerment training.

### Sample Size

The sample size was calculated according to the number of cases needed to identify an effect on health-related quality of life. Because the outcome measure SF-12 has rarely been used in intervention studies among the general population, the SF-36 [18,19] was used for the sample size calculation. Calculated effect sizes range (Cohen's D [20]) from 0.58 (which can be considered 'medium' according to effect size conventions) to 0.96 (considered large) [18,21]. Because of the cluster randomization design, a certain loss of efficiency associated with cluster randomization relative to individual randomization was taken into account [22]. An effect size of 0.40 was considered to be the lower boundary of a 'medium' effect size [20]. This effect size can be detected with a power (1-β) of 0.80 and a two-tailed alpha of 0.05 with two groups of 100. Taking a loss to follow up of about 10% into account, we need to recruit a total of 220 participants.

### Primary outcome measures

#### Work ability

Work ability is measured with the Work Ability Index [4]. This widely used index measures self-assessed work ability and consists of seven items. For the purpose of this study, only three of the seven items were considered relevant and are thus measured. These three items are 1) perceived work ability in general, 2) perceived work ability in relation to physical demands and 3) perceived work ability in relation to mental demands. Different studies have shown that the validity and reliability of Work Ability Index are acceptable to good [23,24].

#### Health-related quality of life

In this study, health-related quality of life is measured with the SF-12 [25,26]. The SF-12 includes items referring to mental health as well as physical health. The following eight dimensions are included: physical functioning, role limitations due to physical problems, bodily pain, general health perceptions, vitality, social functioning, role limitations due to emotional problems, and mental health [27]. Different studies have shown that the validity and reliability of the SF-12 are adequate [26,27].

### Secondary outcome measures

#### Need for recovery

Need for recovery is measured with an existing Dutch questionnaire on the Experience and Evaluation of Work (Dutch abbreviation: VBBA) [28,29], which has shown to be valid and reliable (0.86) [30,31]. The scale consists of eleven dichotomous items (yes/no), representing short-term effects of a working day.

#### Physical workload and musculoskeletal symptoms

Questions about the physical workload are based on questions used for the Periodical Health Screenings survey in the construction industry. In the Netherlands, this survey is widely used and common among construction workers who participate in the Periodical Health Screening.

Data on musculoskeletal symptoms is assessed by means of the Dutch Musculoskeletal Questionnaire (DMQ) [32,33]. In this study, the workers are asked about their symptoms during the last three months and during the past seven days. To provide similarity between the scales, the scale of the seven days period has been adjusted to the two-point scale (yes/no) of the three months.

#### Psychosocial workload

The psychosocial workload is measured by the Dutch version of the Job Content Questionnaire [34,35]. Two constructs of the psychosocial workload (supervisor support and co-worker support) are selected to be measured. These scales have shown moderate to good reliability (0.65-0.81) [36].

#### Awareness, attitude, self-efficacy and social norms

Awareness, attitude, self-efficacy and social norms about reducing physical workload, improving recovery and increasing influence at the worksite are measured to provide insight into the working mechanism of the worksite intervention. Questions about these determinants are formulated based on a structure of questions often used in the health promotion research [37,38].

#### Work engagement

Work engagement is measured with the 12-item questionnaire Utrecht Work Engagement Scale [39]. This scale consists of three dimensions: vigor, dedication and absorption. The psychometric qualities of this scale have been proven to be good [40].

#### The ability and motivation to continue working until the retirement age

Questions about working until the retirement age are assessed by four questions based on the Netherlands Working Conditions Survey [41]. The workers are asked until which age they think they are able and motivated to work. Additionally, they are asked if (physically or mentally) less heavy work can contribute to continue their working life until the age of 65.

### Other variables

#### Sociodemographic and anthropometric data

At baseline, sociodemographic data such as gender, age, level of highest education, working hours per week and anthropometric data such as body height and body weight are assessed.

### Process evaluation

Besides the effect evaluation, a process evaluation is conducted based on the six aspects of Steckler and Linnan (2002): context (organizational and environmental characteristics that affect the intervention), recruitment (sources and procedures used to recruit companies and construction workers), reach (attendance rates of construction workers), dose delivered (the amount of intervention components actually delivered by the trainers), dose received (the extent to which employees use materials or components recommended by the program) and fidelity (the extent to which the intervention was delivered as planned) [42]. In addition, satisfaction (the extent to which the workers were satisfied with the program) is measured. Three of these aspects (context, recruitment and reach) are evaluated by data that is collected in logs since the start of the project in January 2008. Dose delivered and dose received are assessed by checklists completed by the trainers. The remaining aspects, namely fidelity and satisfaction, are obtained by (1) logs from the trainers, (2) questionnaires at three and six months after the start of the intervention, and (3) interviews with supervisors and employees.

### Economic evaluation

An economic evaluation will be conducted alongside the trial and include a cost-benefit analysis and a cost-effectiveness analysis. Both analyses will be performed from a company perspective. The time horizon is 12 months, similar to the trial. A cost-benefit analysis will be carried out to compare the intervention costs with the monetary benefits due to productivity loss. A cost-effectiveness analysis will be conducted for the primary outcomes measures (work ability and health-related quality of life). In the cost-effectiveness analyses, all costs (i.e. costs of the intervention and costs due to productivity loss) will be included and will be compared to the effect on health-related quality of life and work ability. Intervention costs include costs for the development of the intervention as well as the implementation of the intervention (e.g., costs of trainings, video, working hours). Productivity loss (i.e. sick leave and productivity) will be measured with the productivity and disease questionnaire (PRODISQ) [44] and the World Health Organization Health and Work Performance Questionnaire (HPQ) [[45], 46].

### Statistical analyses

Analyses regarding the effectiveness of the primary outcomes and secondary outcomes will be performed after three and six months (short term) and twelve months (long term) by means of multilevel analyses. Multilevel analyses take clustering of observations of workers within the same department into account, as well as repeated measurements within one worker [43]. Due to randomization at the department level, the data will be analyzed at three levels: (1) time, (2) worker and (3) department. Both crude and adjusted linear and logistic regression analyses will be performed. The multilevel analyses using the follow-up measurement (i.e. 3 months) as dependent variable will be adjusted for possible confounding factors such as education and working hours. These variables will also be checked for effect modification. The effect of the intervention at six months and twelve months will be analyzed using all three follow-up measurements (i.e. 3, 6 and 12 months) and will also be adjusted for possible confounders [44]. Effect modification will also be checked again.

For the cost-benefit analysis, the difference in mean intervention costs between the two study groups will be compared to the difference in mean benefits due to sick leave reduction between the two study groups using bias-corrected and accelerated bootstrapping. Confidence intervals (95%) will then be obtained. For the cost-effectiveness analysis, the difference in mean costs (i.e., intervention costs and reduced benefits due to sick leave) between the two study groups will be compared to the difference in mean effects between the two study groups. Cost-effectiveness ratios will be calculated by dividing the difference between the mean total costs between the two study groups by the difference in the mean effects between the study groups. Confidence intervals (95%) will again be obtained by bias corrected and accelerated bootstrapping. For both outcome measures (i.e. health-related quality of life and work ability), cost-effectiveness ratios will be plotted on a cost-effectiveness plane. Acceptability curves will be calculated, showing the probability that the guideline is cost-effective at a specific ratio. Furthermore, sensitivity analyses will be performed to assess the robustness of the results.

All statistical analyses will be performed according to an intention-to-treat principle. In addition, protocol analyses will be conducted for those groups that actually completed the intervention protocol.

## Discussion

This paper presents the design of a randomized controlled trial to investigate the effectiveness of a multi-component worksite prevention program. The content of the intervention consist of two preventive training sessions of a physical therapist, a Rest-Break tool, and two empowerment training sessions.

To our knowledge, this is the first study that will evaluate a multi-component intervention in the construction industry that targets both the individual capacities as well as the work environment. As work ability is a multidimensional concept, such a worksite prevention program seems potentially effective in improving the work ability. Moreover, outcome measures (e.g., work ability, health-related quality of life) will be evaluated which might predict a healthier working life among construction workers.

A strength of the current study is that the evaluation of the intervention will not only give insight into the (cost-)effectiveness, but also into the process of the intervention. The process evaluation aims to describe (1) the reach of the program, (2) the initial expectations and satisfaction of the participating construction workers and (3) the intention of participating companies to further implement the intervention program in the future. Due to time limitations, process evaluations are infrequently conducted in the field of worksite prevention or health promotion and are rarely compared to the outcomes of the study [42,45]. Results of the process evaluation are very relevant as they may provide insight into the working mechanisms of the intervention, and into process factors influencing the outcomes, e.g., was the program intended as planned, and what was the satisfaction with the different components. Moreover, and even more important, the process evaluation will provide information to improve implementation of the program in the future.

A limitation of the current study is that intervention consists of several components and that the RCT is two-armed (control versus intervention), which does not allow separate evaluation of each component of the intervention. As a consequence, eventual effectiveness of the program can only be attributed to the entire program. However, the process evaluation will focus on the entire program as well as on the separate components and will therefore qualitatively gain insight into the working mechanisms of the different components of the intervention.

This intervention may benefit workers as well as employers. If the intervention proves to be effective, the construction worker will benefit from this by an improved health and a healthier working environment and, as such, will contribute to the prolongation of their working life. As a consequence, employers may benefit from having healthier workers in terms of a reduced sick leave and a higher productivity. If this program proves to be cost-effective, the protocol will be made available to all companies in the construction industry as well as for companies in other sectors with high physical work demands.

## Competing interests

The authors declare that they have no competing interests.

## Authors' contributions

CJ and BB wrote the initial study protocol and were involved in preparations for the study. KOH designed the intervention and was responsible for drafting the paper. All authors commented on the draft versions. All authors have read and approved the final version of the manuscript.

## Pre-publication history

The pre-publication history for this paper can be accessed here:

http://www.biomedcentral.com/1471-2458/10/336/prepub
